# Anesthetic management of a patient with methylmalonic acidemia: a case report

**DOI:** 10.1186/s40981-018-0209-7

**Published:** 2018-10-01

**Authors:** Yuta Uemura, Nami Kakuta, Katsuya Tanaka, Yasuo M. Tsutsumi

**Affiliations:** Department of Anesthesiology, Tokushima University, 3-18-15, Kuramoto, Tokushima, 770-8503 Japan

**Keywords:** Methylmalonic acidemia, Methylmalonyl coenzyme A mutase, Metabolic acidosis

## Abstract

**Background:**

Methylmalonic acidemia (MMA) is a metabolic disorder of organic acids and is characterized by the accumulation of methylmalonic acids.

**Case presentation:**

The patient was a 19-year-old female diagnosed with severe MMA at 3 days of age, who was scheduled for renal replacement therapy. Preoperatively, there was no evidence of metabolic acidosis or electrolyte abnormalities. Glucose was administered preoperatively following a 6-h fast. Anesthesia was administered using thiamylal, remifentanil, rocuronium, and sevoflurane. After tracheal intubation, the patient underwent an ultrasound-guided bilateral rectus sheath block with ropivacaine. A drop in blood sugar level was treated with 5% glucose. Extubation was performed after intravenous administration of sugammadex.

**Conclusions:**

We report the anesthetic management of a patient with MMA using a combination of general anesthesia and rectus sheath block.

## Background

Methylmalonic acidemia (MMA) is a metabolic disorder of organic acids in which methylmalonic acids accumulate due to inactivation of methylmalonyl coenzyme A mutase (MCM). MMA is characterized by metabolic acidosis and hyperammonemia and can lead to complications, including mental retardation, seizures, and renal failure. In the present study, we report MMA in a patient undergoing continuous ambulatory peritoneal dialysis catheter placement under the combination of general anesthesia and rectus sheath block.

## Case presentation

The patient was a 19-year-old female with a height of 163.5 cm and a weight of 21.5 kg. She was born after 38 weeks of gestation and weighed 2624 g without asphyxia, but was diagnosed as having severe MMA at 3 days of age due to deterioration of general status, and the presence of metabolic acidosis and hyperammonemia. Complicating her cerebral palsy, she was repeatedly hospitalized due to acidosis and seizures. Since 2009, her renal function gradually worsened, and in 2017, plasma creatinine (Cr) was found to be above 4.0 mg/dl. In 2018, the patient was admitted to the emergency room due to a deterioration of general status from infection, and an operation for a continuous ambulatory peritoneal dialysis catheter placement was scheduled.

Preoperative venous blood gas analysis did not indicate metabolic acidosis: pH, 7.432; PCO_2_, 42.1 mmHg; PO_2_, 38.2 mmHg; and base excess (BE), 2.9 mEq/l. Blood results showed anemia and renal failure: hemoglobin (Hb), 6.1 g/dl; blood urea nitrogen (BUN), 56.0 mg/dl; Cr, 4.04 mg/dl; and estimated glomerular filtration rate (eGFR), 13 ml/min/L.

General anesthesia was combined with a peripheral nerve block. No premedication was administered. After the patient entered the operating room, electrocardiography, saturation of percutaneous oxygen, and noninvasive arterial pressure were monitored. Anesthesia was induced using thiamylal (4.6 mg/kg), remifentanil (0.4 mcg/kg/min), and rocuronium (0.9 mg/kg). After tracheal intubation, the patient underwent an ultrasound-guided bilateral rectus sheath block with 0.2% ropivacaine (30 ml). Anesthesia was maintained with a gas mixture of oxygen and air (FiO_2_ 0.4), 1.0 minimum alveolar anesthetic concentration sevoflurane (1.2–2%), and remifentanil (0.2–0.4 mcg/kg/min).

Intraoperative arterial blood gas values were as follows: pH, 7.369; pCO_2_, 32.9 mmHg; pO_2_, 182 mmHg; BE, − 5.9 mEq/l; Na, 140 mmol/l; K, 3.8 mmol/l; and Cl, 104 mmol/l. Glucose preparation was used to prevent hypoglycemia while maintaining a concentration of 100–200 mg/dl to ensure optimal dosing. The preoperative blood sugar level was 113 mg/dl. Hypotonic fluid (2.5% glucose) was used in the perioperative period. Blood sugar level declined to 82 mg/dl during surgery, and 5% glucose was administered. The level was 120 mg/dl at the end of the surgery. To avoid perioperative accumulation of methylmalonic acid, the temperature was maintained above 36.0 °C with a heating device, and clindamycin was administered intravenously. Throughout the surgery, hemodynamic parameters were stable, and the operation was over without using vasopressor agents. Extubation was performed after intravenous administration of sugammadex (4 mg/kg). The duration of surgery was 2 h and 10 min, and that of anesthesia was 3 h and 30 min. Postoperatively, the patient did not experience pain at rest; however, she experienced mild pain during movement. The patient did not use any rescue analgesic agents in the postoperative period. Additionally, no evidence of metabolic acidosis or hyperammonemia (NH_3_, 29 μg/dl) was observed. Continuous ambulatory peritoneal dialysis was started 10 days after the surgery, and the patient was discharged after 36 days.

## Discussion

MMA is an autosomal inherited disease with various symptoms and ensuing metabolic acidosis, which is characterized by the accumulation of methylmalonic acids due to inactivation of the MCM. MCM is present in the metabolic pathway of four types of amino acids (valine, isoleucine, methionine, and threonine), cholesterol, and odd chain fatty acids (OCFAs). Viability of patients with MMA improves with the progression of treatment, and MMA is capable of being diagnosed early by newborn mass screening. The frequency is 1 in 80,000 [1].

In patients with MMA, anesthetic management is important to prevent acute metabolic failure. Since hypoglycemia worsens metabolic acidosis by protein catabolism sthenia, glucose loading should be avoided. Preoperative fasting should be as short as possible; some studies have reported 6 h as an optimal fasting time [2]. If necessary, glucose may be administered during preoperative fasting. On the other hand, surgical stress can also cause protein catabolism sthenia; therefore, depth of anesthesia should be maintained. Metabolic acidosis should be actively corrected with sodium bicarbonate in cases where the condition did not exist preoperatively. Since some studies have reported metabolic acidosis or ventricular tachycardia with hypokalemia during anesthetic management in patients with MMA [3], arterial blood gas analysis was performed in the present study. Although acidemia was observed, the values did not indicate metabolic acidosis or electrolyte abnormalities. We also administered glucose during surgery due to decreased blood sugar levels.

Selecting appropriate anesthetic drugs is also important. Nitrous oxide should not be used because it interferes with adenosylcobalamin [4]. In general, propofol should also not be administered to patients with MMA. One study reported that propofol use was associated with deterioration of metabolic failure during general anesthesia in a living liver transplantation [2]. Another study has suggested that propofol may be safe for continuous intravenous administration, which was used for sedation during procedures such as magnetic resonance imaging [5]. A general preparation of propofol consists of 1% propofol, 10% soybean oil, 2.25% glycerol, and 1.2% purified egg lecithin [6]. Naturally occurring soybean oil and egg lecithin predominantly consist of 16- and 18-carbon chain fatty acids with minimal-to-none amounts of OCFAs [7]. Based on this viewpoint, the use of propofol appears to be safe in patients with MMA. However, we consider this controversial due to the possibility of the existence of mild subclinical metabolic acidosis alleviated by glucose administration. Furthermore, as our patient had complications associated with end-stage renal failure, we used thiamylal and sevoflurane instead of propofol. Aryl propionic acid, nonsteroidal anti-inflammatory drugs (NSIADs), derivatives of ibuprofen, loxoprofen, and flurbiprofen axetil were avoided because they metabolize into methylmalonic acid. A rectus sheath block was performed with ropivacaine to provide postoperative analgesia for midline incisions. Sugammadex was used to reverse the effects of neuromuscular blocking agents and was shown to be efficacious and safe.

Anesthetic management of patients with MMA needs to be tailored based on an assessment of the pathophysiological condition. Herein, we reported a case of successful anesthetic management of a patient with MMA undergoing continuous ambulatory peritoneal dialysis catheter placement using a combination of general anesthesia and rectus sheath block.

## Abbreviations

ALT: Alanine aminotransferase
AST: Aspartate aminotransferase
BE: Base excess
BUN: Blood urea nitrogen
Cr: Creatinine
eGFR: Estimated glomerular filtration rate
Hb: Hemoglobin
MCM: Methylmalonyl coenzyme A mutase
MMA: Methylmalonic acidemia
NSAIDs: Nonsteroidal anti-inflammatory drugs
